# Immunopathogenic Mechanisms and Novel Immune-Modulated Therapies in Rheumatoid Arthritis

**DOI:** 10.3390/ijms20061332

**Published:** 2019-03-16

**Authors:** Shyi-Jou Chen, Gu-Jiun Lin, Jing-Wun Chen, Kai-Chen Wang, Chiung-Hsi Tien, Chih-Fen Hu, Chia-Ning Chang, Wan-Fu Hsu, Hueng-Chuen Fan, Huey-Kang Sytwu

**Affiliations:** 1 Department of Pediatrics, Tri-Service General Hospital, National Defense Medical Center, No. 325, Section 2, Chenggong Rd., Neihu District, Taipei City 114, Taiwan; pedneuchen@hotmail.com (S.-J.C.); tien.amoebia@msa.hinet.net (C.-H.T.); caperhu@gmail.com (C.-F.H.); Lizy0529@hotmail.com (C.-N.C.); kisetsu1110@gmail.com (W.-F.H.); fanhuengchuen@yahoo.com.tw (H.-C.F.); 2 Department of Microbiology and Immunology, National Defense Medical Center, No. 161, Section 6, MinChuan East Road, Neihu, Taipei City 114, Taiwan; 3 Department of Pediatrics, Penghu Branch of Tri-Service General Hospital, National Defense Medical Center, No. 90, Qianliao, Magong City, Penghu County 880, Taiwan; 4 Graduate Institute of Medical Sciences, National Defense Medical Center, No. 161, Section 6, MinChuan East Road, Neihu, Taipei City 114, Taiwan; 5 Department of Biology and Anatomy, National Defense Medical Center, No. 161, Section 6, MinChuan East Road, Neihu, Taipei City 114, Taiwan; lingujiun@mail.ndmctsgh.edu.tw; 6 Graduate Institute of Life Sciences, National Defense Medical Center, No. 161, Section 6, MinChuan East Road, Neihu, Taipei City 114, Taiwan; jiwechbmo@gmail.com; 7 School of Medicine, National Yang-Ming University, No. 155, Section 2, Linong Street, Taipei City 112, Taiwan; kcwangtpe@gmail.com; 8 Department of Neurology, Cheng Hsin General Hospital, No. 45, Cheng Hsin St., Pai-Tou, Taipei City 112, Taiwan; 9 Department of Pediatrics, Tungs’ Taichung MetroHarborHospital, No. 699, Section 8, Taiwan Blvd., Taichung City 435, Taiwan; 10 National Institute of Infectious Diseases and Vaccinology, National Health Research Institutes, No. 35, Keyan Road, Zhunan, Miaoli County 350, Taiwan

**Keywords:** anticitrullinated peptide antibodies, antirheumatic drug, autoimmune, disease-modifying, immunology, pathology, rheumatoid factor

## Abstract

Rheumatoid arthritis (RA) is a chronic, inflammatory autoimmune disease of unknown etiology. It is characterized by the presence of rheumatoid factor and anticitrullinated peptide antibodies. The orchestra of the inflammatory process among various immune cells, cytokines, chemokines, proteases, matrix metalloproteinases (MMPs), and reactive oxidative stress play critical immunopathologic roles in the inflammatory cascade of the joint environment, leading to clinical impairment and RA. With the growing understanding of the immunopathogenic mechanisms, increasingly novel marked and potential biologic agents have merged for the treatment of RA in recent years. In this review, we focus on the current understanding of pathogenic mechanisms, highlight novel biologic disease-modifying antirheumatic drugs (DMRADs), targeted synthetic DMRADs, and immune-modulating agents, and identify the applicable immune-mediated therapeutic strategies of the near future. In conclusion, new therapeutic approaches are emerging through a better understanding of the immunopathophysiology of RA, which is improving disease outcomes better than ever.

## 1. Introduction

Rheumatoid arthritis (RA) is one of the most widespread chronic immune-mediated inflammatory diseases, with a prevalence of 5–10 cases per 1000 people. It causes joint destruction, pain, and disability [1,2].

### 1.1. Characteristics

The initial symptoms of RA are swelling and pain in the joints of the hands and feet, especially in the metacarpophalangeal, metatarsophalangeal, and proximal interphalangeal joints. Large joints including the elbow, shoulder, ankle, and knee can also be involved [2]. Without adequate treatment, RA progresses to symmetric polyarthritis and destroys the diarthrodial joints of the hands and knees, leading to disability, inability, and mortality.

### 1.2. Current Therapeutics

Patients with RA should receive treatment with disease-modifying antirheumatic drugs (DMARDs). The definition of a DMARD is a medicine that interferes with signs and symptoms of RA, improves physical function, and inhibits the progression of joint damage [3]. Conventional synthetic DMARDs (csDMARDs) have been approved by licensing authorities via empiric clinical observation and have been used for more than 50 years. Methotrexate has been applied for treatment of RA for over 50 years, and even now, methotrexate is the most important of the csDMARDs. If intolerance, contraindications, adverse effects, or inadequate response occur in patients with RA treated with csDMARDs such as methotrexate, then a biologic DMARD and a targeted synthetic DMARD (tsDMARD) have superior efficacy when combined with methotrexate or another csDMARDs, compared with individual use [2]. Currently, IL-6R antibodies and JAK inhibitors are the most efficacious of the biologic DMARDs [4].

### 1.3. Limitations and Unmet Medical Needs

There are still unmet needs in RA treatment; full or stringent remission is not typical, nor is remission usually sustained without continuing treatment, which should now be the priority of research efforts [5]. Another concern is that biologic DMARDs and tsDMARDs are costly.

In this review, we focus on the current understanding of the immunopathogenic mechanisms that cause dysregulation of the inflammatory process leading to structural damage of bone and cartilage in patients with RA. Accordingly, understanding the immune-pathogenic mechanism is pivotal to the development of novel immune-mediated therapies.

## 2. Part I: Immunopathogenic Mechanisms in RA

In the inflammatory process of RA, the cascade responses of innate and adaptive immunity are the essential immune-pathogenic mechanisms [6]. This development is driven by a plethora of inflammatory cytokines and autoantibodies and is sustained by epigenetic changes in fibroblast-like synoviocytes, supporting further inflammation [7,8]. In the intermediate course, large numbers of different immune cells, including neutrophils, granulocytes, macrophages, B-cells, and T-cells invade the synovial membrane and fluid. This invasion results in tremendous releases of cytokines, chemokines, autoantibodies, and reactive oxidative stress (ROS) in the synovial membrane and space, leading to joint destruction. The serological hallmark of the disease is the presence of a high-titer of rheumatoid factor and anticitrullinated peptide antigen and antibodies (ACPAs) [9,10]. Additionally, Vande Walle et al. confirmed that the pathology of RA is strongly related to increased Nlrp3 inflammasome activation in vivo [11]. We discuss this complex mechanism further.

### 2.1. Role of Innate and Adaptive Immune Cells

#### Primary Immune Cells: Macrophages, Neutrophils, and Dendritic Cells in RA

Synovial membrane inflammation reflects consequent immune activation and is characterized by leukocyte invasion by innate immune cells such as monocytes, macrophages, dendritic cells, neutrophils, and adaptive immune cells including Th1, Th2, and Th17 cells, B-cells, and plasma cell lineages [5,12,13].

Both macrophages and neutrophils belong to the subset of phagocytes, which play the first defensive role against pathogens [12,14]. Macrophages contribute to the modulation of the immune response, which initiates immune-mediated inflammation leading to autoimmune disorders [15]. According to their microenvironment, macrophages can be divided into two distinct subsets with different physiological functions, one is proinflammatory subtype (M1), and the other is anti-inflammatory subtype (M2) [16,17].

Several proinflammatory cytokines such as IL-6, TNF-α, and IFN-γ are regulated at the transcriptional level and secreted through the endoplasmic reticulum/Golgi pathway. Interestingly, other proinflammatory cytokines including IL-1β and IL-18, are formed as cytosolic precursors, and their secretion is controlled by inflammatory caspases (caspase-1, -4, and -5) in humans [18]. These caspases are activated within cytosolic multimolecular complexes named inflammasomesin the milieu of the macrophage [19]. These intracellular inflammasomes that induce inflammatory responses in macrophages are activated by different types of ligands, leading tothe induction of inflammatory responses. The hallmark of inflammatory responses is the activation of inflammasomes—multiprotein oligomers containing intracellular pattern recognition receptors and inflammatory effectors—such as caspase recruitment domain (ASC) and pro-caspase-1and subsequently IL-1β andIL-18 is secreted from active macrophagein caspase-1-dependent manner [20]. 

And inflammasomes are classifiedinto three types: (1) ‘canonical inflammsomes’, such as nucleotide-bindingoligomerization domain (NOD)-like receptors (NLRs); (2) absentin melanoma 2 (AIM2) inflammasomes; (3): ‘non-canonicalinflammasomes’, such as caspase-4, -5, and -11 [21]. Remarkably, the nucleotide-binding oligomerization domain (NOD)-like receptor family pyrin domaincontaining 3 (NLRP3) inflammasone is emerging as an important factor in the inflammatory process of RA [19]. 

NLRP3 inflammasomes are highly activated in the infiltration of monocytes and macrophages in synovia but not in fibroblast-like synoviocytes from either RA patients or mice with collagen-induced arthritis (CIA). This activation pattern suggests a pathogenic role for NLRP3 inflammasomes in RA. The activation of NLRP3 inflammasomes was correlated with disease activity and IL-17A concentration in RA sera. Knockdown of NLRP3 suppressed Th17 differentiation MCC950, a selective NLRP3 inhibitor, had proven therapeutic effects in CIA in a murine RA model. MCC950-treated mice with CIA revealed significantly less severe joint inflammation and bone destruction. NLRP3 inflammasome activation in the synovia was significantly inhibited by MCC950, with reduced production of interleukin (IL)-1β [22]. Accordingly, the NLRP3 inflammasome could be a potential therapeutic target for the treatment of RA.

It is notable that the regulatory potential of caspase-11 in inflammatory responses during RA pathogenesis has been focused recently [20]. SinceLacey et al. disclosed the role of caspase-11 and its downstream effectors on inflammatory responses and infectious condition in the model of bacteria—induced inflammatory arthritis via caspase-11 knockout mice and proved that caspase-11 and caspase-1 induced proinflammatory cytokine production and joint inflammation in bacteria-infected arthritis mice, and delayed joint inflammation was observed in caspase-11 knockout mice alternatively. In addition, these results suggest that caspase-11 inflammasome in an IL-18-dependent manner induces the inflammatory responses and pathogenesisof joint inflammation in inflammatory arthritis [23]. 

Similar with canonical inflammasomes, non-canonical inflammasomesstimulate caspase-1 activation and GSDMD cleavage through the formation of cell membrane pores and caspase-1-mediated maturation and secretion of IL-1β and IL-18, suggesting that targeting of the non-canonical inflammasomes and their downstream effectors, such as caspase-11, caspase-1, GSDMD, and proinflammatory cytokines, could be considered as potential targets to suppress inflammatory responses, thus treat inflammatory diseases [20].

Given the existing evidence on the regulatory roles of either canonical or non-canonical inflammasomes during inflammatory responses, selective targeting of these inflammasomes by novel pharmacological approaches may potentially be applied clinically to prevent and treat various human inflammatory diseases including RA [20,24].

### 2.2. Dendritic Cells in RA

Dendritic cells in their role as antigen-presenting cells (APCs) are essential in inducing immunity and in mediating immune tolerance. Dendritic cells are now known to influence many different classes of lymphocytes (T, B, and NK cells) and many types of T-cell responses (Th1/Th2/Th17, regulatory T-cells, peripheral T-cell deletion) [25,26]. Dendritic cells have been investigated extensively in RA pathogenesis and have been implicated in RA [25]. The role of dendritic cells has been studied broadly in the pathogenesis of RA [27]. However, it remains unclear whether dendritic cells initiate autoimmunity in this disease [28]. 

Fully mature dendritic cells express high levels of MHC class II, costimulatory markers (CD86), proinflammatory cytokines (IL-12p70, IL-23, and tumor necrosis factor-α (TNF-α)), all of which are required for the efficient induction of T effector cell responses. Furthermore, the expression of chemokine receptors is controlled during the process of dendritic cell maturation, which enables dendritic cell migration toward lymphoid tissues to present antigen to naïve T-cells. For example, CCR5 is expressed on immature dendritic cells, which is down-regulated during cell maturation; alternatively, CCR7 is overexpressed in maturing dendritic cells [29]. On the other hand, specific repressive molecular patterns with immune suppressive compounds reveal a part of the maturation of dendritic cells with tolerogenic properties. These tolerogenic dendritic cells are considered “semi-mature.” They may be phenotypically mature and exhibit high levels of MHC class II and costimulatory molecules but may express co-inhibitory molecules such as programmed death-ligands 1 and, 2, and immunoglobulin-like transcript 3; they may also characteristically produce immunosuppressive molecules including IL-10, TGF-β, and indoleamine 2,3-dioxygenase. Hence, they have plasticity regarding the functional maturation of dendritic cells, and environmental cues are essential for dendritic cells in the maturation process to determine whether they become immunogenic or tolerogenic [30,31,32]. Currently, tolerogenic dendritic cells are under investigation in clinical trials and could be applied clinically for RA treatment in the future [28,33].

#### 2.2.1. T-Cells, B-Cells, and Cytokine Milieu in RA

T-cells also play a critical role in immune-mediated inflammation of RA. As the disease progresses, activated T-cells aggregate in inflamed joints in experimental CIA models of RA [34,35]. Naïve CD4 T helper (Th) cells can differentiate into distinct lineages (Th1, Th2, and Th17) that are characterized by lineage-specific expression of transcription factors and proinflammatory cytokines upon antigenic stimulation [36,37].

Before the era of proven Th17 cells, the imbalance of Th1 and Th2 was considered the central regulatory mechanism of adaptive immunity in autoimmune diseases including RA. Several studies have revealed that Th1 cells are found predominantly in RA joints [38]; alternatively, down-regulation of the Th1 response in experimental arthritis increased the Th2 response [39]. 

Since the discovery of Th17 cells more than one decade ago, their significance in RA has gradually emerged [40]. Human Th17 cell development is regulated by a transcription factor and RAR-related orphan receptor C. These cells express IL-17A, IL-17F, IL-21, IL-22, IL-26, TNF-α, GM-CSF, andCCL20 [41,42], which play specific roles in the immune response and exhibit synergetic effects [13,40]. The increased amounts of Th1, Th2, and Th17 cells are demonstrated [13,43], while that of regulatory T-cells (Tregs) suppresses disease severity in CIA [44]. In addition, Tregs are reduced in the blood of RA patients [45]. The dysregulation of CD4+ and CD8+ T-cells influences the autoimmune progression, depending on the presence of autoreactive Th1 and Th17 CD4+ T-cells, leading to RA immunopathology and disease development [45]. 

#### 2.2.2. B-Cells in RA

Citrullinated antigen-directed B-cells of patients with RA and reacted with citrullinated antigens have a substantial in vitro effect [46]. This citrullinated antigen-directed B-cell response contributes to the initiation and persistence of the inflammatory process. Therefore, anticitrullinated protein antibody (ACPA) response is the primary humoral immune response associated with RA [10,47]. Accordingly, the biologic DMRADs for targeting B-cells was developed as the initial priority that is reviewed in this article.

Abnormal kinetics among immune cells results in an aberrant orchestra of activated T-cells, B-cells, mast cells, neutrophils, macrophages, and access APCs (i.e., dendritic cells), all of which contribute to the cellular immune responses of the RA disease process [48]. 

#### 2.2.3. Immune-Mediated Inflammatory Milieu in RA

The initial effector cells of RA are neutrophils that release high levels of oxidants and cytotoxic products, such as ROS, and inflammatory agents including TNF-α, proteases, phospholipases, defensins, and myeloperoxidase at the site of acute RA in the affected joint. In chronic inflammation of RA, Th17 cells are involved in the induction of tissue inflammation by stimulation from recruited neutrophils. Reciprocally, these activated Th17 cells generate neutrophil chemoattractants such as IL-8 and TNF-α in the joint [49,50,51,52]. Neutrophils in the joint then facilitate the activation of Th17 cells through the secretion of Th17-maintaining chemokines CCL20 and CCL2 [53]. Likewise, neutrophils play a role in the activation of NK cells. The depletion of neutrophils can impair maturation, function, and homeostasis of NK cells [54]. Macrophages, while activated, play another crucial role in the inflammatory course of RA, and these cells, which are highly plastic, can polarize into either the M1 or M2 phenotype; M1 cells secrete proinflammatory cytokines, whereas M2 cells secrete anti-inflammatory cytokines [55,56].

M1 macrophages produce proinflammatory cytokines such as TNFα, IL-1β, IL-6, IL-12, IL-23, and low levels of IL-10 and inflammatory enzymes in the process of promoting acute RA. M1 macrophages also release inflammatory chemokines including CXCL5, CXCL8, CXCL9, CXCL10, and CXCL13 to recruit further leukocytes to the inflammatory site, and these cells produce more IL-1β, TNF-α IL-6, MMP, chemokine receptors, ROS, and inducible nitric oxide synthase in the joint, leading to joint destruction [49,57]. An evolutionary ancient inflammatory proteincalled high mobility group box 1 (HMGB1) rapidly activates APCs and activates innate and adaptive immune responses. This protein has been studied in patients with neuromyelitis optica and multiple sclerosis [58]. The risk factors associated with HMGB1 single nucleotide polymorphisms have been demonstrated in the development of RA disease among the Chinese Han population [59]. Thus, HMGB1 may be an emerging target for RA therapy.

The dominant function of M2 macrophages is anti-inflammation. Thus, M2 macrophages remodel and repair tissue by the production of IL-10, IL-12, and expression of CD163, and CD206, as well as releasing growth factors such as TGF-β and vascular endothelial growth factor (VEGF) during chronic inflammation [60,61]. Calreticulin (CRT), an endoplasmic reticulum residential glycoprotein, plays a crucial role in maintaining intracellular Ca^2+^ homeostasis. Soluble CRT accumulates in the blood of RA patients [62]. In addition, soluble oligomerized CRT could have a pathogenic function in autoimmune diseases through the induction of proinflammatory cytokines (e.g., TNF-α and IL-6) by macrophages via the MAPK-NF-κB signaling pathway [63]. This phenomenon implies soluble CRT has pathologic capability in RA, which could provide a strategy for a new therapeutic approach to RA.

The importance of the crosstalk between T-cells and monocytes in promoting inflammation is growing [64]. Remarkably, in RA, the receptors for IL-17 (IL-17RA and IL-17RC) are found in the synovium and are expressed on CD14+monocytes and macrophages, whereas synoviocytes bind with IL-17 to induce stimulation of further inflammation and production of IL-6 and MMPs in the synovium [61,65]. In addition, monocytes and macrophages from the synovial fluid of the inflamed arthritic joint can promote IL-17 production in CD4^+^ T-cells [66], suggesting that subsequently recruited CD4+T-cells in the rheumatoid joint can develop into a Th17 lineage in association with residential monocytes and macrophages. Consequently, a reciprocal synchronous loop between Th17 cells and monocytes and macrophages enables inflammation [13,67]. Regulatory T-cells (Tregs) are key participants in the regulation of various immune responses. Tregs have the potential to direct macrophages to develop into the M2 phenotype, with the functional and phenotypic characteristics of immune modulators [68]. Tregs express novel surface receptors Tregs such as neuropilin-1, CD83, and G protein-coupled receptor 83, which have advanced our understanding of Treg modulating mechanisms [69]. Thus, to target T-cell-macrophage interactions may have therapeutic potential in RA.

IL-6 and IL6R contribute to IL-6 blockade therapy currently on RA [70,71]. IL-6 is produced by a variety of cells such as endothelial cells, fibroblasts, keratinocytes, chondrocytes, some tumor cells, and immune cells including monocytes, macrophages, T-cells, and B-cells. IL-6 receptor is assembled from two subunits. One is IL-6-specific receptor (IL-6R), and the other is a signal transducer (gp130). Both the subunits exist in membrane-bound and soluble forms, named mIL-6R, sIL-6R, mgp130, and s130, respectively. However, mIL-6R is expressed only on some leukocytes while gp130 is found on many cells in body. IL-6 binds to mIL-6R, and the complex subsequently associates with signal transducing molecule gp130, which induces the activation of downstream signaling events in target cells via Janus kinase. This association leads to classic proinflammatory signaling. Alternatively, sIL-6R, without transmembrane and cytoplasmic regions converts to the anti-inflammatory pathway [8,72]. High levels of IL-6 have been detected in the blood and synovial fluid of most patients with RA. IL-6 facilities neutrophils to secrete ROS and proteolytic enzymes, which augment inflammation and eventually damage joints [73]. IL-6 causes inflammation and joint destruction by acting on neutrophils that secrete reactive oxygen intermediates and proteolytic enzymes. In addition, IL-6 stimulates osteoclast differentiation by activation of either RANKL-dependent or RANKL-independent mechanisms [74]. 

IL-6 enhances production of chemokines such as monocyte chemotactic protein-1 and IL-8 from endothelial cells, mononuclear cells, and fibroblast-like synoviocytes; it also induces adhesion molecules such as ICAM-1 in endothelial cells and induces increased adhesion of monocytes to endothelial cells in RA [75,76]. The synergistic effect of IL-6 with IL-1β and TNF-α stimulate the production of VEGF, which is an essential cytokine in the organization and maintenance of pannus [77]. 

Thus, knowledge and understanding of new and updated immunopathologic mechanisms could elicit the design and discovery of novel therapeutic and immune-modulatory agents to improve the life quality and disease control in RA.

## 3. Part II: Current Immune Target Therapy and on-Going Immune-Modulated Therapy in RA

By definition, DMARDs target inflammatory processes and lessen subsequent damage in diseasessuch asRA [5]. Monoclonal antibodies have been used extensively over the past two decades in clinical trials of RA treatments. TNFα–blocking monoclonal antibodies have been clinically proven and applied in patients with RA. However, the response period is limited [78]. Subsequently, many biologic and immune targeting agents have emerged with therapeutic effects in patients with RA. Furthermore, DMARDs cocktails mixed with several monoclonal antibodies or immune-modulated agents have been pre-clinically or clinically tested for inducing disease remission and maintenance [79]. 

Inspiringly, several biologic agents targeting cytokines and cytokine networks have achieved significant successes in RA treatment. Rituximab, a monoclonal antibody against the CD20 expressed on the surface of B-cells for depletion of B-cell, has been used in RA for more than one decade. Five TNF-α targeting biologic drugs are approved for treatment of RA, including etanercept, infliximab, adalimumab, certolizumab, pegol, and golimumab. Nonetheless, a substantial minority of patients with RA do not respond to these medications, which has necessitated the development of other biologic agents. Currently, tocilizumab, an anti-IL6R mAb; abatacept, a soluble fusion protein that consists of the extracellular domain of cytotoxic T-lymphocyte–associated antigen 4 linked to the modified Fc portion of IgG1, which interferes with T-cell activation; and tofacitinib, a Janus kinase class inhibitor that inhibits intracellular signaling are in clinical use [71]. 

We review the success of these therapeutic agents and potential strategies for RA treatment.

### 3.1. B-Cell Targeting Therapy

The first randomized, double-blind placebo-controlled trial of rituximab was completed in 2004 for patients with long-standing active RA, despite methotrexate treatment, or in combination with either cyclophosphamide or continued methotrexate, with significant improvement [80]. In addition, the efficacy and safety of different rituximab doses plus methotrexate, with or without glucocorticoids, in patients with active RA who did not respond to conventional DMARDs were tried in one clinical study. Either low or high dosages of rituximab were effective and well tolerated [81,82]. One concern is that serum sickness may occur following a first rituximab infusion without recurrence after the second infusion [83].

In patients with RA with an insufficient response to anti-TNF-α therapy, a single course of rituximab with methotrexate provided a significant improvement in disease activity and clinical progression of radiological damage [84]. An open-label prospective study further confirmed that rituximab is a treatment option for patients who do not respond to a single dose of TNF-α inhibitor, particularly for seropositive patients(patients with positive anti-CCP or RF) [85]. 

### 3.2. Anti-TNF-α Therapy

The strategy for blocking TNF-α was introduced to clinical practice at the end of the last century and revolutionized the treatment of RA as well as many other inflammatory conditions. Steeland et al. recently conducted an impressive review of successful anti-RA therapeutics with TNF-inhibitors (TNFi), including etanercept, infliximab, adalimumab, certolizumab, pegol, and golimumab [86]. Infliximab, adalimumab, and golimumab are full-length monoclonal antibodies, and thus, apart from their general TNF-blockage properties, they have Fc-effector activity as well. They can induce antibody-dependent cellular cytotoxicity (ADCC) and trigger the complement pathway leading to cell-dependent cytotoxicity (CDC) and apoptosis of target immune cells. Etanercept, a soluble TNF receptor, contains a truncated Fc-domain without the CH1 domain of IgG1; therefore, the potency of etanercept to induce ADCC and CDC is less than that of monoclonal antibodiessuch asinfliximab [87]. Certolizumab pegol, is a Fab’ fragment, and its structure is incapable of inducing ADCC and CDC; therefore, its functioning mechanism is not dependent on the complement pathway [86,88].

Notably, Nguyen et al. demonstrated that adalimumab, but not etanercept, paradoxically promoted the interaction between monocytes and Tregs isolated from patients with RA. Adalimumab bound to monocyte membrane TNF and surprisingly enhanced its expression and its binding to TNF-RII expressed on Tregs. Consequently, adalimumab expanded functional Foxp3(+) T reg cells capable of suppressing Th17 cells through an IL-2/STAT5-dependent mechanism [89]. 

Total B-cell numbers are reduced in the blood of patients with RA vs. healthy controls but are significantly higher (normal levels) in patients undergoing anti–TNFα therapy. Cardiovascular disease, including heart failure and infections, represent the leading causes of disability and mortality in patients with RA [90]. Patients treated with anti–TNFα antibody alone or with methotrexate seem to be at further risk of severe infection such as tuberculosis [91,92]. As a result, the anti-TNFα treatment is contraindicated in all patients with heart failure and a substantial portion of patients with RA and impaired heart function who do not benefit from the treatment [93]. 

Nonetheless, anti–TNFα therapies are widely and successfully used despite the risk of serious adverse events. Presently, anti-TNF therapies are initiated as the standard-of-care in RA patients when methotrexate treatment fails to provide relief. TNF-inhibitors combined with methotrexate are used in the treatment of 70–80% of RA cases [86,94].

### 3.3. Anti-IL-12/IL-23 Therapy

TGF-β, IL-23, and proinflammatory cytokines function to drive and modulate human Th17 responses in RA [95,96]. Moreover, increased Th17 cell numbers and poor clinical outcomes in RA patients are associated with a genetic variant in the *IL4R* gene [97]. Accordingly, IL-12 and IL-23 are implicated in the pathogenesis of RA and may be considered candidate molecules for immune targeting in RA. Ustekinumab is a human monoclonal antibody targeting the IL-12/23 p40 subunit, which, in clinical trials, has inhibited both IL-12 and IL-23 activity and is effective in relieving moderate-to-severe psoriasis and active psoriatic arthritis [98,99].

Guselkumab is a new monoclonal antibody targeting IL-23 and is effective for psoriasis relief in a clinical trial [100]. The safety and efficacy of ustekinumab and guselkumab were studied in adults with active RA regardless of methotrexate therapy. However, targeting of IL-12/IL-23 p40 (ustekinumab) and IL-23 alone (guselkumab) were not proved yet in RA treatments [101]. 

### 3.4. Anti-IL6 Signaling Therapy

IL-6 is a kind of cytokine with multi-biological functions that include regulation of immune reaction, inflammation, and hematopoietic effects. IL-6 possesses quite a lot of proinflammatory characters, such as stimulating the production of chemokines and adhesion molecules in lymphocytes [4], and increasing neutrophil numbers in the blood [6].

Tocilizumab, humanized anti-IL-6 receptor (IL-6R) monoclonal antibody, is highly efficacious for the treatment of intractable autoimmune inflammatory diseases, including RA and juvenile idiopathic arthritis (JIA) in clinical trials [70]. 

Tofacitinib is a novel, oral Janus kinase (JAK) inhibitor-mediated by JAK1 and JAK3 to regulate STAT1 and STAT3 through the IL-6/gp130/STAT3 signaling pathway. Tofacitinib has been shown to amelirate arthritis symptoms effectively in patients with RA, and oral tofacitinib is Food and Drug Administration (FDA) approved for the treatment of RAand approved by the EMA [74,102]. Moreover, tofacitinib down-regulates the production of proinflammatory cytokines IL-17 and IFN-γ and the proliferation of CD4+ T-cells in patients with RA [103,104].

Global data has shown that patients with RA and inadequate response or intolerance to anti-TNFα therapy can often be effectively managed by switching to a drug with a novel mechanism of action, such as an IL-6R inhibitor [105]. Blockade of IL-6 signaling (via a monoclonal antibody to the IL-6 receptor, tocilizumab) reportedly boosts Tregs and inhibit monocyte IL-6 mRNA expression, inducing monocyte apoptosis [106,107,108,109]. Sarilumab, a fully human monoclonal antibody against IL-6R, has shown efficacy and safety in patients with active RA with an inadequate response to methotrexate in a randomized clinical trial [110,111]. Furthermore, in a phase III clinical trial, sarilumab has shown effectiveness in RA patients with an inadequate response to tumor necrosis factor inhibitor (TNFi). Sarilumab plus csDMARDs significantly decreased circulating biomarkers and synovial inflammation and bone resorption, including C1M, C3M, CXCL13, MMP-3 tRANKL levels, and sICAM-1 [112]. 

Baricitinib is a novel oral, once-daily targeted synthetic DMARD (tsDMARD) that inhibits JAK1 and JAK2. JAK1 and JAK2 are involved in the immunopathogenesis of RA by increasing the turnover of active, phosphorylated STAT1 and STAT3, and preventing chemotaxis toward IL-8. Baricitinib is approved in the FDA, EU and Japan for the treatment of patients with moderate or severe active RA who did not respond well or were intolerant of csDMARD(s) [48,113,114].

### 3.5. Anti-Cytotoxic T-Lymphocyte–Associated Antigen 4 Therapy

CTLA4-immunoglobulin (Ig) (abatacept) is a fusion protein containing components of IgG and cytotoxic T-lymphocyte–associated antigen 4 that inhibit costimulatory signals from APCs distinctively impairing T-cell costimulatory signals by binding to CD80 and CD86 receptors on APCs to target the interaction between monocytes and T-cells and prevent T-cell activation [115]. Abatacept significantly reduced disease severity and enhanced physical function in RA patients who experienced inadequate responses to methotrexate and TNFi [61,116]. Nonetheless, some patients did not respond to abatacept. They had an increased proportion of CD28-cells among CD4+ cells suggesting that CD4+ CD28+ Tfh-like cells could be targets of abatacept. Therefore, the presence of CD4+ CD28−cells may be a potential predictor of abatacept resistance [117]. 

### 3.6. Tolerogenic Dendritic Cells in RA

Tregs play an essential role in maintaining immune tolerance. Restoration of Treg function is a promising target for clinical intervention in autoimmune diseases. One treatment method is reloading the Treg pool in autoimmune patients with functional Tregs, either by treating the patients with drugs that selectively expand the Treg population in vivo or by generating new Tregs ex vivo before infusing them into the patient [118]. The challenge of Treg therapy is how to achieve the expansion of antigen-specific Tregs and how to determine the appropriate antigen(s) to activate the Tregs. One remarkable strategy that is developing is using tolerogenic dendritic cells to induce Tregs that are active against heat-shock proteins (HSPs) ubiquitously expressed in inflamed target tissues [119]. 

Interestingly, HSP 60 is expressed in the synovial membranes of patients with RA, and monoclonal antibodies that recognize mammalian HSP 60 were detected in patients with chronic arthritis. Similar results were noted for the HSP family members HSP 40 and HSP 70 in synovial fluid and on circulating T-cells of patients. The strategy is to pulse tolerogenic dendritic cells with targeted HSP peptides to generate HSP-specific T-cells from dendritic cells with stable tolerogenic function and to induce a regulatory effect on the specific antigen. Thus, such a combination therapy of tolerogenic dendritic cells and HSP peptide therapy could be the optimal solution for autoantigen(s) in autoimmune diseases such as RA [120,121].

Disease-specific ACPAs were found in a large population of RA patients and were strongly associated with HLA-DRB1 risk alleles [5]. Inspiringly, intradermal rheumavax, which is the first tolerogenic dendritic cell therapy in a clinical trial for the treatment of patients with RA with HLA risk genotype-positive and citrullinated peptide-specific autoimmunity, was usually well tolerated and considered safe [122]. 

Lack of vitamin D, especially vitamin D3, is regarded as a critical factor in autoimmune rheumatic disease, including initial disease development, and it is associated with poorer clinical outcomes [123,124]. The second trial of tolerogenic dendritic cells in RA was of dexamethasone and vitamin D3 for tolerogenic dendritic cell generation [125]. The generation of these cells with both dexamethasone and vitamin D3 had a synergistic effect on increasing IL-10 levels [126,127].

## 4. Clinical Trials and the Spotlight for RA in the Future

### 4.1. MicroRNA

MicroRNAs (miRNAs) are small non-coding RNAs that regulate gene expression by modulating the cell transcriptome directly [128,129]. miR-155 is a multifunctional miRNA with high production in immune cells and is essential for the immune response. Conversely, deregulation of miR-155 contributes to the progression of chronic inflammation in RA, and thus, miR-155 has potential as a therapeutic target for the treatment of RA [130]. Visfatin is a newly discovered adipocyte enzyme [131] that promotes the production of IL-6 and TNF-α via the inhibition of miR-199a-5p expression through the ERK, p38, and JNK pathways and is also a potential target for disease biomarker and drug development in inflammatory arthritis [132]. 

### 4.2. PI3Kγ Inhibitors

PI3Kγ mediates the modulation of chemokine-induced migration and enrollment of neutrophils, monocytes, and macrophages in patients with RA [133]. PI3Kγ is considered a potential target in the treatment of RA, though no drug has been developed yet to date [134]. In addition, pathogenic autoantibodies contribute significantly to antibody-initiated inflammation in RA progression. Targeting IgG by glyco-engineering bacterial enzymes to specifically cleave IgG and alter N-linked Fc-glycans or blocking the downstream effector pathways offers a novel opportunity to develop therapeutics for RA treatment in the future [135]. Novel drugs targeting IL-12/IL-23 axis have been proven effective in the treatment of severe psoriasis and psoriatic arthritis (i.e., ustekinumab targeting the IL-12/23 p40 subunit that inhibits IL-12 and IL-23 activity and guselkumab targeting IL-23) [136]. The randomized phase II studies of ustekinumab and guselkumab in RA have shown convincing results, but infection is a primary concern [101]. 

The therapeutic design of TGFβ-transduced mesenchymal stem cells (MSCs) with enhanced immunomodulatory activity in experimental autoimmune arthritis reduces disease severity and modulates T-cell-mediated immune response. Thus, the use of gene-modified MSCs may be an avenue for new therapeutic development for RA treatment [137]. Several types of stem cells, such as hematopoietic stem cells, MSCs, and Tregs, are currently in clinical trials [138]. 

Attractively, novel anti-RA therapeutics had been developed currently such as antagonizing targets to histamine H4, histone deacetylase, LHRH (luteinizing hormone-releasing hormone), cadherin, MMP-9, CX3C ligand 1, and TLR4.

### 4.3. Histamine H4

Histamine H4 receptor (H4R) preserves immune-modulatory and chemotaxic potentials in various immune cells, andclozapine—a HR4 antagonist could protect mice from arthritis [139]. In addition, Yamaura et al. demonstrated that tJNJ77777120 (JNJ)—histamine4receptor (H4R) H4R antagonist exhibits significant anti-inflammatory and anti-arthritic activities in a mouse model of collagen antibody-induced arthritis (CAIA). Suggesting the apparent involvement of H4R antagonism in the pathogenesis and progression of RA and implying that H4R in synovial tissue play a role in cartilage and bone destruction by influencing the secretion of MMP-3 in RA patients [140]. 

### 4.4. Histone Deacetylase (HDAC)

Since histone deacetylase (HDAC) inhibitors repress the production of IL-6 in RA-FLS and macrophages by promoting mRNA decay [141]. Thus, the therapeutic potential of HDAC inhibitors had been investigated in RA and many HDAC inhibitors have been developed, e.g., the pan HDAC inhibitors, such as ITF 2357 and SAHA, inhibit all HDACs, and the selective HDAC inhibitors, such as Tubastatin A and Tubacin, inhibit HDAC6 specifically [142]. 

WhileHDAC3 powerfully regulates STAT1 activity in RA-FLS, indicating HDAC3 as a potential therapeutic target in the treatment of RA and type I IFN-driven autoimmune diseases [143]. 

Moreover, the therapeutic effect of a novel specific HDAC6 inhibitor, CKD-L, compared to the pan HDAC inhibitors, ITF 2357 or Tubastatin A on CIA and Treg cells isolated from RA patients. In the CIA model, CKD-L and Tubastatin A significantly ameliorated the arthritis severity. CKD-L increasedCTLA-4 expression in Foxp3+ T-cells and inhibited the T-cells proliferation in the suppression assay. InRA PBMC, CKD-L significantly increased IL-10, and inhibited TNF-α and IL-1β. These results suggest that CKD-L—a novel HDAC6 inhibitor may have a therapeutic effect of RA in the future [143]. 

### 4.5. Cadherins

Cadherin-11 expressed mainly in the synovial lining and FLS adhered to cadherin-11-Fc are first proved, supporting an important role for cadherin-11 in the specific adhesion of FLS and in synovial tissue organization and behavior in health and RA [144]. Furthermore, Lee et al. demonstrate cadherin-11-deficient mice with a hypoplastic synovial lining that display a disorganized synovial reaction to inflammation and resistant to inflammatory arthritis. Accordingly, synovial cadherin-11 determines the manner of synovial cells in their proinflammatory and destructive tissue effects in inflammatory arthritis [145]. 

In the joint, cadherin-11 is critical for synovial development. In synovial fibroblasts, cell surface cadherin-11 engagement with a recombinant soluble form of the cadherin-11 extracellular binding domain linked to immunoglobulin Fc tail induced MAPK and NF-κB activation, leading to significant IL-6, chemokines, and MMP expression [146]. Currently, a monoclonal antibody againstcadherin-11 is in early phases of clinical trials in patients with RA [147], and the result is expectable.

### 4.6. LHRH (Luteinizing Hormone-Releasing Hormone)

RA symptoms may develop or burst during stimulation of the hypothalamic-pituitary-gonadal axis, such as during the menopausal transition, postpartum, anti-estrogen treatment, or polycystic ovarian syndrome while GnRH and gonadotropin secretion increases [148]. 

GnRH-antagonism—cetrorelix produced rapid anti-inflammatory effects in terms of decreased TNF-α, IL-1β, IL-10, and CRP compared with placebo in RA patients with high gonadotropin levels [149]. Therefore, current developed GnRH-antagonism—cetrorelix has the positive effects in RA that addresses the potential therapeutic candidate in RA patients with high level GnRH.

### 4.7. MMP-9

High expression of transcription factor SOX5 was detected in RA-FLS and MMP-9 expression was inhibited from the knockdown model of SOX5 in CIA mice, suggesting thatSOX5 at least a part plays a pivotal role in mediating migration and invasion of FLS by regulating MMP-9 expression in RA that was confirmed inhibited in the joint tissue and reduced pannus migration and invasion into the cartilage [150].

Exposure of monocytes/macrophages to tocilizumab, etanercept or abatacept is resulted in a significant decrease of the PMA-induced superoxide anion production. The expression of PPARγ was significantly increased only by tocilizumab, while etanercept was the only one able to significantly reduce MMP-9 gene expression and inhibit the LPS-induced MMP-9 activity in monocytes. An uneven production of proinflammatory cytokines and MMP-9 in diseased articular joint tissues probably is affected by IL-17 through interacting with the macrophages in the rheumatoid synovium [151]. Thus, to block MMP-9 may be a potential strategy for developing novel therapeutics in RA.

### 4.8. CX3C Ligand 1

CX3CL1 is the member of t CX3C chemokines also named Fractalkine. CX3CL1 plays a role in monocyte chemotaxis and angiogenesis in the rheumatoid synoviumin RA. Increased MMP-2 production is detected from synovial fibroblasts upon CX3CL1 stimulation in vitro, suggesting a proinflammatory role of this Th1-type chemokine in RA [152]. Synergistic up-regulation of CX3CL1 protein also was observed after treatment with IL-1β and IFN-γ. The production of lung fibroblast-derived CX3CL1 were obviously reduced by specific inhibitors of the STAT-1 transcription factor, supporting the hypothesis that lung fibroblasts are an important cellular source of CX3CL1 and may contribute to causing pulmonary inflammation and fibrosis [153]. Recently, a clinical trial of an anti-CX3CL1 monoclonal antibody for the treatment of RA had been inaugurated in Japan. The multiple roles of CX3CL1 are in the pathogenesis of RA, to block CXCL1 may have a potential as a therapeutic target for this disease [154]. 

### 4.9. Toll-Like Receptors (TLRs)-TLR4

ACPA precede the onset of clinical and subclinical RA., ACPA fine profiling has the potential to identify RA patients with a predominantly TLR4-driven pathotype. Thus, TLR4 ligands may drive pathogenic processes of ACPA based on their target specificity in RA and thus address the potential therapeutic benefit when neutralizing TLR4 in the disease of RA [155]. Neutralization of TLR4 signaling was designed by using NI-0101, which is a TLR4 antagonism in terms of therapeutic and specific antibody to target TLR4. NI-0101-aTLR4 inhibition in an ex vivo model of RA pathogenesis can significantly amend cytokines release including IL1, IL-6, IL-8 and TNF-α.

Pharmacological inhibition of TLR4 and NF-κB activation blocked the HMGB1-dependent up-regulation of HIF-1α mRNA expression and its activity and HMGB1 stimulated expression of EGF, and inhibition of HIF-1α attenuated HMGB1-induced VEGF [156]. 

DFMG attenuates the activation of macrophages induced by co-culture with LPC-injured HUVE-12 cells via the TLR4/MyD88/NF-κB signaling pathway [157]. Predictably, the therapeutic design of TLR4 inhibition may be assumed as a therapeutic candidate for development in the treatment of RA soon or later.

### 4.10. Inflammasomes

Both hydroxychloroquine and VX740 are the potential candidates for the treatment of RA in the near future through modulation of inflammasomes [158]. Hydroxychloroquine (complex formationof the inflammasome) represses overexpression of TLR leading to inhibit the secretion of TNF-α. VX740—the inhibitor of caspase-1 inhibits CARD overexpression in RA and Decrease NLRP-3 and downstream proinflammatorycytokines. These novel approaches light on another therapeutic strategy in RA.

## 5. Conclusions

### 5.1. Challenge: Lessons from Targeted Interventions

Serious opportunistic infections rarely occur in long-term rituximab therapy. Nonetheless, patients and physicians must be aware that such opportunistic infections can occur. One example is the reactivation of the John Cunningham virus leading to progressive multifocal leukoencephalopathy, which has been reported in patients with autoimmune diseases. Patients must be informed of the risk of such adverse events with rituximab therapy [159,160]. Additionally, long-term rituximab therapy is related to hypogammaglobulinemia. Thus, basal immunoglobulin levels should be determined and carefully monitored to judge how rituximab therapy should be managed in light of IgG levels [161]. Additionally, late-onset neutropenia is a potential rituximab-related adverse event; thus, neutrophil levels should be carefully monitored [81,162].

A few biologic drugs such as TNF-α, IL6R, and CD20 inhibitors, can cause complications of neutropenia. Combination therapy of abatacept and G-CSF reduced such neutropenia [163]. 

The safety profile of oral tofacitinib seems acceptable, although some severe adverse effects have been observed, including serious and opportunistic infections (including tuberculosis and herpes zoster), malignancies, and cardiovascular events, which require strict monitoring irrespective of the duration of tofacitinib administration. As an oral drug, tofacitinib offers an alternative to subcutaneous or intravenous administration and should be recognized as a more convenient way of drug administration [163].

In RA, current immune-modulated therapies fail to maintain long-term physiological regulation in drug-free remission. Nonetheless, autologous conditioned tolerogenic dendritic cells with HSP-derived peptide antigen(s) could be used to restore immune tolerance. These treatments could either promote tolerance in pathologic T-cells or stimulate disease-suppressing Tregs in a dissimilarmanner [120]. 

### 5.2. Potential Targets for RA in the Future

Since a better understanding of the pathophysiology of RA, new therapeutic approaches are emerging (Table 1). Many novel approaches appear to have good therapeutic potential in the challenges of developing new treatments for RA [104]. We have summarized these potential targets in Table 2 including targets of microRNA (miR-155 and Visfatin) [164], PI3Kγ for chemokine-induced migration, histamine 4 receptor (H4R) through blocking H4R in synovial tissue to prevent the destruction cartilage and bone, histone deacetylase (HDAC), cadherin-11, LHRH (luteinizing hormone-releasing hormone), CX3CL1, TLR4, and activation of inflammasomes. (summarized in Table 2)

There is worth in studyingthe mechanisms of pathogenesis of RA and understanding causes of therapeutic failure in RA—for example, IL-1, IL-12, IL-17, IL-20, IL-21, IL-23, anti-CD4, anti-BAFF, and inhibitors of p38-MAPK and SYK [5]. The ultimate goal is todevelop cause-oriented, curative therapies; however, this willnot be easily achievable without better understanding of theexact cause(s) of RA.Nevertheless, through the development of early and precious diagnostic approaches and novel therapeuticsthat will provide more précised and efficient treatment of RA in the near future.

## Figures and Tables

**Table 1:** Summary of novel treatment for RA.

Drug/Delivery	Target	Mechanism	Immune-Modulation
	**Target CD80/CD86 receptor on T cells**		
Abatacept (Orencia®) /Intravenous delivery	Fc-fusion protein of the extracellular domain of human CTLA-4	block the binding reaction between CD80/CD86 and CD28, a costimulatory signal required for complete activation of T cells and inhibition of TNFα, and IFNγ production by activated T cells.	TNFα inducing the expression of innate cytokines IL-1β, IL-6 and IL-8, resulting in the rapid recruitment of neutrophils upon exposure to infection is blocked by and inhibition of TNFα, and IFNγ production by preveting T cells activation
	**Antagonist of IL-1**		
Anakinra (Kineret®) / Subcutaneous injection	Block the reaction of IL-1 binding to IL-1RI	Block the reaction of IL-1 binding to IL-1RI resulting in intracellular signal transduction	Lessen the IL-1 effect on increasing the synovial fibroblast cytokine, chemokine, iNOS, PGs and MMPs release.
	**IL-6 receptor monoclonal antibody**		
Sarilumab (Kevzara®)/Subcutaneous injection	a human IgG1 antibody, specifically binds to soluble and membrane-bound IL-6R (sIL-6Ra and mIL-6Ra)	Inhibit IL-6-mediated signalling involving ubiquitous signal transducing gp130 and STAT3	Interference the activator RANKL dependent or RANKL independent mechanismand also block the synergismwith IL-1β and TNF-α in producing VEGF
	**IL-12/IL-23 antibodies**		
Ustekinumab (STELARA®)/ Subcutaneous injection	targeting the IL-12/23 p40 subunit, inhibiting both IL-12 and IL-23 activities	Bind to the IL-12 and IL-23, cytokines and down modulate lymphocyte function, including Th 1 and Th17 cell subsets	Inhibit IL-12-mediated signaling to reduce intracellular phosphorylation of STAT4 and STAT6 proteins, and impair the responses including cell surface molecule expression, NK cell activities and cytokine production, i.e., IFNγ
Guselkumab (Tremfya®)/ Subcutaneous injection	Specific targeting IL-23	Bind to IL-23 and repress induction of Th17 cell subsets IL-23, mainly produced by dendritic cells, macrophages	Block IL-23 target cells via either an IL-17-dependent or an IL-17-independent mechanism and decrease IL-23 secretion and inpair activation of producing Th17 cells via IL-23R and reduce cytokine such as IL-17 or IL-22
	**JAK inhibitor**		
Tofacitinib (Xeljanz®)/ ORAL	the first-in-class JAK inhibitor, block JAK1 and JAK3 factor.	Interference the binding of IL-6 to the IL-6Rα/gp130 complex, STAT proteins	Tofacitinib block the pathway of JAK/STAT activation; due to JAK/STAT activation by IL-7 *versus IL-6 or GM-CSF* and the recruited to the cytokine/receptor complex
Baricitinib (Olumiant®) Decernotinib ORAL	Selective JAK1 and JAK2 inhibitor	Block with intracellular signal transduction , facilate the turnover of active, phosphorylated STAT1 and STAT3	inhibition of cytokine (IL-6) or thrombopoietin and reduce the expression of pathogenic Th1 and Th17 and prevent chemotaxis towards IL-8
Filgotinib/ORAL	selective JAK1 inhibitor	Block with intracellular signal transduction , facilate the turnover of active, phosphorylated STAT1	decrease IL-1β, IL-6, TNFα and MMP1 and MMP3, inhibit immune cell trafficking (CXCL10, ICAM-1 and VCAM-1) and VEGF. Also significantly decrease Th1 and Th17 cell subset differentiation and activity
Rheumavax®/Intradermal injection	first-in-human trial for the treatment of RA generated tDC by NF-κB inhibition	InhibitNF-κB and prevent DC maturation to reduce the expression of CD40 and HLA-DR ( a class II MHC molecule)	confers tolerogenic properties to DC including induction of T-cell anergy elevation of B220+ CD11c− B cells with a subpopulation of B-regulatory cells (Bregs)
Pulsing tlDCs with HSP peptides /Intravenous delivery with with HSP loaded tDCs	HSP 40 (dnaJB1): dnaJP1 HSP 60 (HspD1): DiaPep277 HSP 70 (HspA9): mB29a	Induce disease-suppressive regulatory T cells	induce IL-10 production and TGF-β

**Table 2:** Summary of potential targets for RA.

Drug or Compound	Target	Potential Mechanism	Immune-Modulation
Developing inhibitor of miR-155	miR-155	regulatory functions on the expression of genes by modulating the cell transcriptome directly	Inhibit TLR/cytokine receptor pathways and suppress the production of TNF, IL-1β, IL-6, and chemokines CCR7
Developing inhibitor of visfatin	Visfatin	Upregulation of miR-199a-5p expression through modulation of the ERK, p38, and JNK pathways	Decrease the production of IL-6 and TNF-α
Developing inhibitor of PI3Kγ	PI3Kγ	modulation of chemokine-induced migration	Control enrollment of inflammatory cells (i.e., neutrophils, monocytes, and macrophages)
andclozapine tJNJ77777120 (JNJ)	Histamine 4 receptor (H4R)	Block H4R in synovial tissue to prevent the destruction cartilage and bone	Immune-modulatory effect and repression of chemotaxic potentials by influencing the secretion of MMP-3
the pan HDAC inhibitors: ITF 2357 and SAHA HDAC6 inhibitors: Tubastatin A, Tubacin, and CKD-L	histone deacetylase (HDAC)	repress the production of IL-6 in RA FLS and macrophages by promoting mRNA decay	CKD-L increased CTLA-4 expression in Foxp3+ T cells and inhibited the T cells proliferation in the suppression assay. CKD-L significantly increased IL-10, and inhibited TNF-α and IL-1β
a monoclonal antibody against cadherin-11	cadherin-11	Block the reaction of engagement with a recombinant soluble form of the cadherin-11 extracellular binding domain linked to immunoglobulin Fc tail induced MAPK and NF-κB activation in SFL	Suppression the production of IL-6, chemokines, and MMP expression in SFL
GnRH-antagonism—cetrorelix	LHRH (luteinizing hormone-releasing hormone)	rapid anti-inflammatory effects	decreased TNF-α, IL-1β, IL-10, and CRP
knock-down model of SOX5	Block the MMP-9	Inhibit high expression of transcription factor SOX5 in RA-FLS	repressed IL-17 through interacting with the macrophages
anti-CX3CL1 monoclonal antibody	CX3CL1	Block monocyte chemotaxis and angiogenesis	Decreased MMP-2
NI-0101, a TLR4 antagonism	TLR4	block the HMGB1-dependent upregulation of HIF-1α mRNA expression	amend cytokines release including IL1, IL-6, IL-8 and TNF-α.
Hydroxychloroquine (complex formation of the inflammasome)	TLR overexpression	Inflammasome priming mechanism	Potential decreased TNF-α
VX 740	Caspace-1	Inhibit CARD8 overexpression	Decrease NLRP-3 and dwonstream cytokines

## Abbreviations

ACPA	anticitrullinated protein antibody
TNFi	tumor necrosis factor inhibitor
APC	antigen-presenting cell
CRT	calreticulin
CIA	collagen-induced arthritis
DMARDs	disease-modifying antirheumatic drugs
csDMARD	conventional synthetic DMARD
tsDMARD	targeted synthetic DMARD
JAK	Janus kinase
MMP	matrix metalloproteinase
M1	type 1 macrophage
M2	type 2 macrophage
miRNA	microRNA
MSC	mesenchymal stem cell
NLRP3	nucleotide-binding oligomerization domain (NOD)-like receptor family pyrin domain containing 3
RA	rheumatoid arthritis
RANKL	receptor activator of NF-kappa B ligand;
ROS	reactive oxygen species
TNF-α	tumor necrosis factor-α
Th	T helper
TGF-β	tumor growth factor-β
Treg	regulatory T-cell
VEGF	vascular endothelial growth factor
